# Draft Genome Sequence of *Tepidibacter mesophilus* Strain JCM 16806^T^ Isolated from Soil Polluted by Crude Oil in China

**DOI:** 10.1128/genomeA.01308-17

**Published:** 2017-11-22

**Authors:** Chol Gyu Lee, Masahiro Yuki, Toshiya Iida, Kazuhiro Nakaho, Moriya Ohkuma

**Affiliations:** a Japan Collection of Microorganisms, RIKEN BioResource Center, Tsukuba, Ibaraki, Japan; b Institute of Vegetable and Floriculture Science, National Agriculture and Food Research Organization, Tsu, Mie, Japan

## Abstract

Here, we report the draft genome sequence of *Tepidibacter mesophilus* strain JCM 16806^T^, which was isolated from an oil field. It is composed of 3,310,272 bp and contains 3,160 protein-coding genes, 8 5S rRNAs, 3 16S rRNAs, and 69 tRNAs.

## GENOME ANNOUNCEMENT

We characterize here three species of bacteria belonging to the genus *Tepidibacter*, *T. thalassicus* (1), *T. formicigenes* strain DSM 15518 (2), and *T. mesophilus* strain JCM 16806^T^ (3). The former two species are thermophilic and isolated from hydrothermal vents, but the latter one is mesophilic and isolated from soil contaminated by crude oil. *T. mesophilus* strain JCM 16806^T^ is anaerobic, aerotolerant, endospore forming, and fermentative; optimal growth occurred at 28 to 32°C (3).

Genomic DNA was extracted from three independent liquid cultures using the NucleoSpin tissue kit (Macherey and Nagel, Düren, Germany) according to the manufacturer’s protocols. The genome of *T. mesophilus* strain JCM 16806^T^ was sequenced using the Illumina MiSeq sequencing platform (Illumina, San Diego, CA, USA) with a paired-end strategy (600 cycles). SPAdes version 3.8.1 (4) was used to perform a *de novo* assembly of the reads. A total of 564,185 reads were assembled into 110 contigs with an *N*_50_ length of 100,223 bp and the largest length of 358,450 bp. This assembly resulted in a draft genome sequence of 3,310,272 bp with 33.8× redundancy and a G+C content of 28.8%. The draft genome sequence size and G+C content of *T. mesophilus* are larger than those of *T. thalassicus* and *T. formicigenes* strain DSM 15518 (2,234,370 and 2,639,825 bp, 27.9 and 27.7%, respectively).

The genome annotation was analyzed using the Rapid Annotations using Subsystems Technology (RAST) server (5, 6). The genome annotation identified 3,160 protein-coding genes and 80 RNA genes (8 5S rRNAs, 3 16S rRNAs, and 69 tRNAs). The annotation assigned these genes into 366 subsystems, with a maximum number of genes associated with amino acids and derivatives (16.64%).

### Accession number(s).

The *T. mesophilus* genome was deposited in DDBJ/EMBL/GenBank under the accession numbers BDQY01000001 to BDQY01000110.
